# Research design: the methodology for interdisciplinary research framework

**DOI:** 10.1007/s11135-017-0513-8

**Published:** 2017-04-27

**Authors:** Hilde Tobi, Jarl K. Kampen

**Affiliations:** 1 Biometris, Wageningen University and Research, PO Box 16, 6700 AA Wageningen, The Netherlands; 2 Statua, Dept. of Epidemiology and Medical Statistics, Antwerp University, Venusstraat 35, 2000 Antwerp, Belgium

**Keywords:** Research design, Interdisciplinarity, MIR, Education, Consultancy, Multidisciplinary collaboration

## Abstract

Many of today’s global scientific challenges require the joint involvement of researchers from different disciplinary backgrounds (social sciences, environmental sciences, climatology, medicine, etc.). Such interdisciplinary research teams face many challenges resulting from differences in training and scientific culture. Interdisciplinary education programs are required to train truly interdisciplinary scientists with respect to the critical factor skills and competences. For that purpose this paper presents the Methodology for Interdisciplinary Research (MIR) framework. The MIR framework was developed to help cross disciplinary borders, especially those between the natural sciences and the social sciences. The framework has been specifically constructed to facilitate the design of interdisciplinary scientific research, and can be applied in an educational program, as a reference for monitoring the phases of interdisciplinary research, and as a tool to design such research in a process approach. It is suitable for research projects of different sizes and levels of complexity, and it allows for a range of methods’ combinations (case study, mixed methods, etc.). The different phases of designing interdisciplinary research in the MIR framework are described and illustrated by real-life applications in teaching and research. We further discuss the framework’s utility in research design in landscape architecture, mixed methods research, and provide an outlook to the framework’s potential in inclusive interdisciplinary research, and last but not least, research integrity.

## Introduction

Current challenges, e.g., energy, water, food security, one world health and urbanization, involve the interaction between humans and their environment. A (mono)disciplinary approach, be it a psychological, economical or technical one, is too limited to capture any one of these challenges. The study of the interaction between humans and their environment requires knowledge, ideas and research methodology from different disciplines (e.g., ecology or chemistry in the natural sciences, psychology or economy in the social sciences). So collaboration between natural and social sciences is called for (Walsh et al. 1975).

Over the past decades, different forms of collaboration have been distinguished although the terminology used is diverse and ambiguous. For the present paper, the term interdisciplinary research is used for (Aboelela et al. 2007, p. 341):

any study or group of studies undertaken by scholars from two or more distinct scientific disciplines. The research is based upon a conceptual model that links or integrates theoretical frameworks from those disciplines, uses study design and methodology that is not limited to any one field, and requires the use of perspectives and skills of the involved disciplines throughout multiple phases of the research process.

Scientific disciplines (e.g., ecology, chemistry, biology, psychology, sociology, economy, philosophy, linguistics, etc.) are categorized into distinct scientific cultures: the natural sciences, the social sciences and the humanities (Kagan 2009). Interdisciplinary research may involve different disciplines within a single scientific culture, and it can also cross cultural boundaries as in the study of humans and their environment.

A systematic review of the literature on natural-social science collaboration (Fischer et al. 2011) confirmed the general impression of this collaboration to be a challenge. The nearly 100 papers in their analytic set mentioned more instances of barriers than of opportunities (72 and 46, respectively). Four critical factors for success or failure in natural-social science collaboration were identified: the paradigms or epistemologies in the current (mono-disciplinary) sciences, the skills and competences of the scientists involved, the institutional context of the research, and the organization of collaborations (Fischer et al. 2011). The so-called “paradigm war” between neopositivist versus constructivists within the social and behavioral sciences (Onwuegbuzie and Leech 2005) may complicate pragmatic collaboration further.

It has been argued that interdisciplinary education programs are required to train truly interdisciplinary scientists with respect to the critical factor skills and competences (Frischknecht 2000) and accordingly, some interdisciplinary programs have been developed since (Baker and Little 2006; Spelt et al. 2009). The overall effect of interdisciplinary programs can be expected to be small as most programs are mono-disciplinary and based on a single paradigm (positivist-constructivist, qualitative-quantitative; see e.g., Onwuegbuzie and Leech 2005). We saw in our methodology teaching, consultancy and research practices working with heterogeneous groups of students and staff, that most had received mono-disciplinary training with a minority that had received multidisciplinary training, with few exceptions within the same paradigm. During our teaching and consultancy for heterogeneous groups of students and staff aimed at designing interdisciplinary research, we built the framework for methodology in interdisciplinary research (MIR). With the MIR framework, we aspire to contribute to the critical factors skills and competences (Fischer et al. 2011) for social and natural sciences collaboration. Note that the scale of interdisciplinary research projects we have in mind may vary from comparably modest ones (e.g., finding a link between noise reducing asphalt and quality of life; Vuye et al. 2016) to very large projects (finding a link between anthropogenic greenhouse gas emissions, climate change, and food security; IPCC 2015).

In the following section of this paper we describe the MIR framework and elaborate on its components. The third section gives two examples of the application of the MIR framework. The paper concludes with a discussion of the MIR framework in the broader contexts of mixed methods research, inclusive research, and other promising strains of research.

## The methodology in interdisciplinary research framework

### Research as a process in the methodology in interdisciplinary research framework

**Fig. 1:**
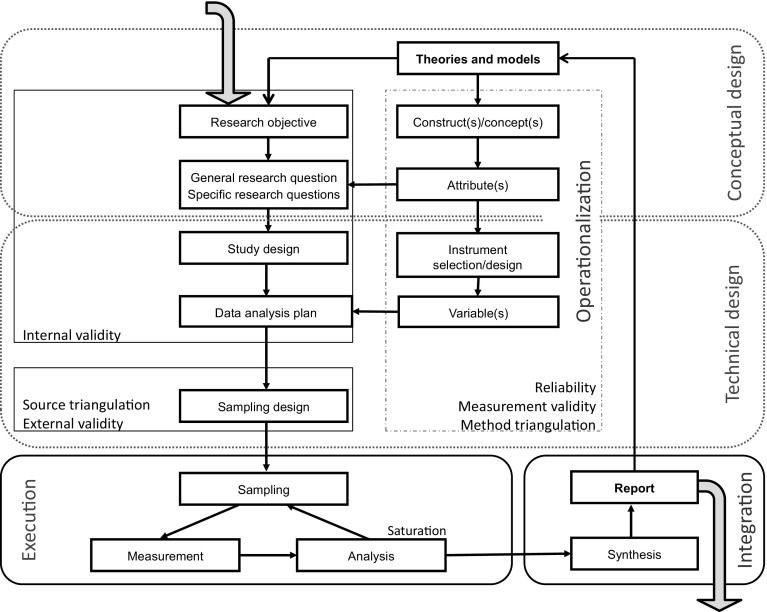
The Methodology of Interdisciplinary Research framework

The Methodology for Interdisciplinary Research (MIR) framework was built on the process approach (Kumar 1999), because in the process approach, the research question or hypothesis is leading for all decisions in the various stages of research. That means that it helps the MIR framework to put the common goal of the researchers at the center, instead of the diversity of their respective backgrounds. The MIR framework also introduces an agenda: the research team needs to carefully think through different parts of the design of their study before starting its execution (Fig. 1). First, the team discusses the conceptual design of their study which contains the ‘why’ and ‘what’ of the research. Second, the team discusses the technical design of the study which contains the ‘how’ of the research. Only after the team agrees that the complete research design is sufficiently crystalized, the execution of the work (including fieldwork) starts.

Whereas the conceptual and technical designs are by definition interdisciplinary team work, the respective team members may do their (mono)disciplinary parts of fieldwork and data analysis on a modular basis (see Bruns et al. 2017: p. 21). Finally, when all evidence is collected, an interdisciplinary synthesis of analyses follows which conclusions are input for the final report. This implies that the MIR framework allows for a range of scales of research projects, e.g., a mixed methods project and its smaller qualitative and quantitative modules, or a multi-national sustainability project and its national sociological, economic and ecological modules.

### The conceptual design

Interdisciplinary research design starts with the “conceptual design” which addresses the ‘why’ and ‘what’ of a research project at a conceptual level to ascertain the common goals pivotal to interdisciplinary collaboration (Fischer et al. 2011). The conceptual design includes mostly activities such as thinking, exchanging interdisciplinary knowledge, reading and discussing. The product of the conceptual design is called the “conceptual frame work” which comprises of the research objective (what is to be achieved by the research), the theory or theories that are central in the research project, the research questions (what knowledge is to be produced), and the (partial) operationalization of constructs and concepts that will be measured or recorded during execution. While the members of the interdisciplinary team and the commissioner of the research must reach a consensus about the research objective, the ‘why’, the focus in research design must be the production of the knowledge required to achieve that objective the ‘what’.

With respect to the ‘why’ of a research project, an interdisciplinary team typically starts with a general aim as requested by the commissioner or funding agency, and a set of theories to formulate a research objective. This role of theory is not always obvious to students from the natural sciences, who tend to think in terms of ‘models’ with directly observable variables. On the other hand, students from the social sciences tend to think in theories with little attention to observable variables. In the MIR framework, models as simplified descriptions or explanations of what is studied in the natural sciences play the same role in informing research design, raising research questions, and informing how a concept is understood, as do theories in social science.

Research questions concern concepts, i.e. general notions or ideas based on theory or common sense that are multifaceted and not directly visible or measurable. For example, neither food security (with its many different facets) nor a person’s attitude towards food storage may be directly observed. The operationalization of concepts, the transformation of concepts into observable indicators, in interdisciplinary research requires multiple steps, each informed by theory. For instance, in line with particular theoretical frameworks, sustainability and food security may be seen as the composite of a social, an economic and an ecological dimension (e.g., Godfray et al. 2010).

As the concept of interest is multi-disciplinary and multi-dimensional, the interdisciplinary team will need to read, discuss and decide on how these dimensions and their indicators are weighted to measure the composite interdisciplinary concept to get the required interdisciplinary measurements. The resulting measure or measures for the interdisciplinary concept may be of the nominal, ordinal, interval and ratio level, or a combination thereof. This operationalization procedure is known as the port-folio approach to widely defined measurements (Tobi 2014). Only after the research team has finalized the operationalization of the concepts under study, the research questions and hypotheses can be made operational. For example, a module with descriptive research questions may now be turned into an operational one like, what are the means and variances of X1, X2, and X3 in a given population? A causal research question may take on the form, is X (a composite of X1, X2 and X3) a plausible cause for the presence or absence of Y? A typical qualitative module could study, how do people talk about X1, X2 and X3 in their everyday lives?

### The technical design

Members of an interdisciplinary team usually have had different training with respect to research methods, which makes discussing and deciding on the technical design more challenging but also potentially more creative than in a mono-disciplinary team. The technical design addresses the issues ‘how, where and when will research units be studied’ (study design), ‘how will measurement proceed’ (instrument selection or design), ‘how and how many research units will be recruited’ (sampling plan), and ‘how will collected data be analyzed and synthesized’ (analysis plan). The MIR framework provides the team a set of topics and their relationships to one another and to generally accepted quality criteria (see Fig. 1), which helps in designing this part of the project.

Interdisciplinary teams need be pragmatic as the research questions agreed on are leading in decisions on the data collection set-up (e.g., a cross-sectional study of inhabitants of a region, a laboratory experiment, a cohort study, a case control study, etc.), the so-called “study design” (e.g., Kumar 2014; De Vaus 2001; Adler and Clark 2011; Tobi and van den Brink 2017) instead of traditional ‘pet’ approaches. Typical study designs for descriptive research questions and research questions on associations are the cross-sectional study design. Longitudinal study designs are required to investigate development over time and cause-effect relationships ideally are studied in experiments (e.g., Kumar 2014; Shipley 2016). Phenomenological questions concern a phenomenon about which little is known and which has to be studied in the environment where it takes place, which calls for a case study design (e.g., Adler and Clark 2011: p. 178). For each module, the study design is to be further explicated by the number of data collection waves, the level of control by the researcher and its reference period (e.g., Kumar 2014) to ensure the teams common understanding.

Then, decisions about the way data is to be collected, e.g., by means of certified instruments, observation, interviews, questionnaires, queries on existing data bases, or a combination of these are to be made. It is especially important to discuss the role of the observer (researcher) as this is often a source of misunderstanding in interdisciplinary teams. In the sciences, the observer is usually considered a neutral outsider when reading a standardized measurement instrument (e.g., a pyranometer to measure incoming solar radiation). In contrast, in the social sciences, the observer may be (part of) the measurement instrument, for example in participant observation or when doing in-depth interviews. After all, in participant observation the researcher observes from a member’s perspective and influences what is observed owing to the researcher’s participation (Flick 2006: p. 220). Similarly in interviews, by which we mean “a conversation that has a structure and a purpose determined by the one party—the interviewer” (Kvale 2007: p. 7), the interviewer and the interviewee are part of the measurement instrument (Kvale and Brinkmann 2009: p. 2). In on-line and mail questionnaires the interviewer is eliminated as part of the instrument by standardizing the questions and answer options. Queries on existing data bases refer to the use of secondary data or secondary analysis. Different disciplines tend to use different bibliographic data bases (e.g., CAB Abstracts, ABI/INFORM or ERIC) and different data repositories (e.g., the European Social Survey at europeansocialsurvey.org or the International Council for Science data repository hosted by www.pangaea.de).

Depending on whether or not the available, existing, measurement instruments tally with the interdisciplinary operationalisations from the conceptual design, the research team may or may not need to design instruments. Note that in some cases the social scientists’ instinct may be to rely on a questionnaire whereas the collaboration with another discipline may result in more objective possibilities (e.g., compare asking people about what they do with surplus medication, versus measuring chemical components from their input into the sewer system). Instrument design may take on different forms, such as the design of a device (e.g., pyranometer), a questionnaire (Dillman 2007) or a part thereof (e.g., a scale see DeVellis 2012; Danner et al. 2016), an interview guide with topics or questions for the interviewees, or a data extraction form in the context of secondary analysis and literature review (e.g., the Cochrane Collaboration aiming at health and medical sciences or the Campbell Collaboration aiming at evidence based policies).

Researchers from different disciplines are inclined to think of different research objects (e.g., animals, humans or plots), which is where the (specific) research questions come in as these identify the (possibly different) research objects unambiguously. In general, research questions that aim at making an inventory, whether it is an inventory of biodiversity or of lodging, call for a random sampling design. Both in the biodiversity and lodging example, one may opt for random sampling of geographic areas by means of a list of coordinates. Studies that aim to explain a particular phenomenon in a particular context would call for a purposive sampling design (non-random selection). Because studies of biodiversity and housing obey the same laws in terms of appropriate sampling design for similar research questions, individual students and researchers are sensitized to commonalities of their respective (mono)disciplines. For example, a research team interested in the effects of landslides on a socio-ecological system may select for their study one village that suffered from landslides and one village that did not suffer from landslides that have other characteristics in common (e.g., kind of soil, land use, land property legislation, family structure, income distribution, et cetera).

The data analysis plan describes how data will be analysed, for each of the separate modules and for the project at large. In the context of a multi-disciplinary quantitative research project, the data analysis plan will list the intended uni-, bi- and multivariate analyses such as measures for distributions (e.g., means and variances), measures for association (e.g., Pearson Chi square or Kendall Tau) and data reduction and modelling techniques (e.g., factor analysis and multiple linear regression or structural equation modelling) for each of the research modules using the data collected. When applicable, it will describe interim analyses and follow-up rules. In addition to the plans at modular level, the data analysis plan must describe how the input from the separate modules, i.e. different analyses, will be synthesized to answer the overall research question. In case of mixed methods research, the particular type of mixed methods design chosen describes how, when, and to what extent the team will synthesize the results from the different modules.

Unfortunately, in our experience, when some of the research modules rely on a qualitative approach, teams tend to refrain from designing a data analysis plan before starting the field work. While absence of a data analysis plan may be regarded acceptable in fields that rely exclusively on qualitative research (e.g., ethnography), failure to communicate how data will be analysed and what potential evidence will be produced posits a deathblow to interdisciplinarity. For many researchers not familiar with qualitative research, the black box presented as “qualitative data analysis” is a big hurdle, and a transparent and systematic plan is a sine qua non for any scientific collaboration. The absence of a data analysis plan for all modules results in an absence of synthesis of perspectives and skills of the disciplines involved, and in separate (disciplinary) research papers or separate chapters in the research report without an answer to the overall research question. So, although researchers may find it hard to write the data analysis plan for qualitative data, it is pivotal in interdisciplinary research teams.

Similar to the quantitative data analysis plan, the qualitative data analysis plan presents the description of how the researcher will get acquainted with the data collected (e.g., by constructing a narrative summary per interviewee or a paired-comparison of essays). Additionally, the rules to decide on data saturation need be presented. Finally, the types of qualitative analyses are to be described in the data analysis plan. Because there is little or no standardized terminology in qualitative data analysis, it is important to include a precise description as well as references to the works that describe the method intended (e.g., domain analysis as described by Spradley 1979; or grounded theory by means of constant-comparison as described by Boeije 2009).

### Integration

To benefit optimally from the research being interdisciplinary the modules need to be brought together in the integration stage. The modules may be mono- or interdisciplinary and may rely on quantitative, qualitative or mixed methods approaches. So the MIR framework fits the view that distinguishes three multimethods approaches (quali–quali, quanti–quanti, and quali–quant).

Although the MIR framework has not been designed with the intention to promote mixed methods research, it is suitable for the design of mixed methods research as the kind of research that calls for both quantitative and qualitative components (Creswell and Piano Clark 2011). Indeed, just like the pioneers in mixed methods research (Creswell and Piano Clark 2011: p. 2), the MIR framework deconstructs the package deals of paradigm and data to be collected. The synthesis of the different mono or interdisciplinary modules may benefit from research done on “the unique challenges and possibilities of integration of qualitative and quantitative approaches” (Fetters and Molina-Azorin 2017: p. 5). We distinguish (sub) sets of modules being designed as convergent, sequential or embedded (adapted from mixed methods design e.g., Creswell and Piano Clark 2011: pp. 69–70). Convergent modules, whether mono or interdisciplinary, may be done parallel and are integrated after completion. Sequential modules are done after one another and the first modules inform the latter ones (this includes transformative and multiphase mixed methods design). Embedded modules are intertwined. Here, modules depend on one another for data collection and analysis, and synthesis may be planned both during and after completion of the embedded modules.

### Scientific quality and ethical considerations in the design of interdisciplinary research

A minimum set of jargon related to the assessment of scientific quality of research (e.g., triangulation, validity, reliability, saturation, etc.) can be found scattered in Fig. 1. Some terms are reserved by particular paradigms, others may be seen in several paradigms with more or less subtle differences in meaning. In the latter case, it is important that team members are prepared to explain and share ownership of the term and respect the different meanings. By paying explicit attention to the quality concepts, researchers from different disciplines learn to appreciate each other’s concerns for good quality research and recognize commonalities. For example, the team may discuss measurement validity of both a standardized quantitative instrument and that of an interview and discover that the calibration of the machine serves a similar purpose as the confirmation of the guarantee of anonymity at the start of an interview.

Throughout the process of research design, ethics require explicit discussion among all stakeholders in the project. Ethical issues run through all components in the MIR framework in Fig. 1. Where social and medical scientists may be more sensitive to ethical issues related to humans (e.g., the 1979 Belmont Report criteria of beneficence, justice, and respect), others may be more sensitive to issues related to animal welfare, ecology, legislation, the funding agency (e.g., implications for policy), data and information sharing (e.g., open access publishing), sloppy research practices, or long term consequences of the research. This is why ethics are an issue for the entire interdisciplinary team and cannot be discussed on project module level only.

## The MIR framework in practice: two examples

### Teaching research methodology to heterogeneous groups of students

#### Institutional context and background of the MIR framework

Wageningen University and Research (WUR) advocates in its teaching and research an interdisciplinary approach to the study of global issues related to the motto “To explore the potential of nature to improve the quality of life.” Wageningen University’s student population is multidisciplinary and international (e.g., Tobi and Kampen 2013). Traditionally, this challenge of diversity in one classroom is met by covering a width of methodological topics and examples from different disciplines. However, when students of various programmes received methodological education in mixed classes, students of some disciplines would regard with disinterest or even disdain methods and techniques of the other disciplines. Different disciplines, especially from the qualitative respectively quantitative tradition in the social sciences (Onwuegbuzie and Leech 2005: p. 273), claim certain study designs, methods of data collection and analysis as their territory, a claim reflected in many textbooks. We found that students from a qualitative tradition would not be interested, and would not even study, content like the design of experiments and quantitative data collection; and students from a quantitative tradition would ignore case study design and qualitative data collection. These students assumed they didn’t need any knowledge about ‘the other tradition’ for their future careers, despite the call for interdisciplinarity.

To enhance interdisciplinarity, WUR provides an MSc course mandatory for most students, in which multi-disciplinary teams do research for a commissioner. Students reported difficulties similar to the ones found in the literature: miscommunication due to talking different scientific languages and feelings of distrust and disrespect due to prejudice. This suggested that research methodology courses ought help prepare for interdisciplinary collaboration by introducing a single methodological framework that 1) creates sensitivity to the pros and challenges of interdisciplinary research by means of a common vocabulary and fosters respect for other disciplines, 2) starts from the research questions as pivotal in decision making on research methods instead of tradition or ontology, and 3) allows available methodologies and methods to be potentially applicable to any scientific research problem.

#### Teaching with MIR—the conceptual framework

As a first step, we replaced textbooks by ones refusing the idea that any scientific tradition has exclusive ownership of any methodological approach or method. The MIR framework further guides our methodology teaching in two ways. First, it presents a logical sequence of topics (first conceptual design, then technical design; first research question(s) or hypotheses, then study design; etc.). Second, it allows for a conceptual separation of topics (e.g., study design from instrument design). Educational programmes at Wageningen University and Research consistently stress the vital importance of good research design. In fact, 50% of the mark in most BSc and MSc courses in research methodology is based on the assessment of a research proposal that students design in small (2-4 students) and heterogeneous (discipline, gender and nationality) groups. The research proposal must describe a project which can be executed in practice, and which limitations (measurement, internal, and external validity) are carefully discussed.

Groups start by selecting a general research topic. They discuss together previously attained courses from a range of programs to identify personal and group interests, with the aim to reach an initial research objective and a general research question as input for the conceptual design. Often, their initial research objective and research question are too broad to be researchable (e.g., Kumar 2014: p. 64; Adler and Clark 2011: p. 71). In plenary sessions, the (basics of) critical assessment of empirical research papers is taught with special attention to the ‘what’ and ‘why’ section of research papers. During tutorials students generate research questions until the group agrees on a research objective, with one general research question that consists of a small set of specific research questions. Each of the specific research questions may stem from a different discipline, whereas answering the general research question requires integrating the answers to all specific research questions.

The group then identifies the key concepts in their research questions, while exchanging thoughts on possible attributes based on what they have learnt from previous courses (theories) and literature. When doing so they may judge the research question as too broad, in which case they will turn to the question strategies toolbox again. Once they agree on the formulation of the research questions and the choice of concepts, tasks are divided. In general, each student turns to the literature he/she is most familiar with or interested in, for the operationalization of the concept into measurable attributes and writes a paragraph or two about it. In the next meeting, the groups read and discuss the input and decide on the set-up and division of tasks with respect to the technical design.

#### Teaching with MIR—the technical framework

The technical part of research design distinguishes between study design, instrument design, sampling design, and the data analysis plan. In class, we first present students with a range of study designs (cross sectional, experimental, etc.). Student groups select an appropriate study design by comparing the demands made by the research questions with criteria for internal validity. When a (specific) research question calls for a study design that is not seen as practically feasible or ethically possible, they will rephrase the research question until the demands of the research question tally with the characteristics of at least one ethical, feasible and internally valid study design.

While following plenary sessions during which different random and non-random sampling or selection strategies are taught, groups start working on their sampling design. The groups make two decisions informed by their research question: the population(s) of research units, and the requirements of the sampling strategy for each population. Like many other aspects in research design, this can be an iterative process. For example, suppose the research question mentioned “local policy makers,” which is too vague for a sampling design. Then the decision may be to limit the study to “policy makers at the municipality level in the Netherlands” and adapt the general and the specific research questions accordingly. Next, the group identifies whether a sample design needs to focus on diversity (e.g., when the objective is to make an inventory of possible local policies), representativeness (e.g., when the objective is to estimate prevalence of types of local policies), or people with particular information (e.g., when the objective is to study people having experience with a given local policy). When a sample has to representative, the students must produce an assessment of external validity, whereas when the aim is to map diversity the students must discuss possible ways of source triangulation. Finally, in conjunction with the data analysis plan, students decide on the sample size and/or the saturation criteria.

When the group has agreed on their population(s) and the strategy for recruiting research units, the next step is to finalize the technical aspects of operationalisation i.e. addressing the issue of exactly how information will be extracted from the research units. Depending on what is practically feasible qua measurement, the choice of a data collection instrument may be a standardised (e.g., a spectrograph, a questionnaire) or less standardised (e.g., semi-structured interviews, visual inspection) one. The students have to discuss the possibilities of method triangulation, and explain the possible weaknesses of their data collection plan in terms of measurement validity and reliability.

#### Recent developments

Presently little attention is payed to the data analysis plan, procedures for synthesis and reporting because the programmes differ on their offer in data analysis courses, and because execution of the research is not part of the BSc and MSc methodology courses. Recently, we have designed one course for an interdisciplinary BSc program in which the research question is put central in learning and deciding on statistics and qualitative data analysis. Nonetheless, during the past years the number of methodology courses for graduate students that supported the MIR framework have been expanded, e.g., a course “From Topic to Proposal”; separate training modules on questionnaire construction, interviewing, and observation; and optional courses on quantitative and qualitative data analysis. These courses are open to (and attended by) PhD students regardless of their program. In Flanders (Belgium), the Flemish Training Network for Statistics and Methodology (FLAMES) has for the last four years successfully applied the approach outlined in Fig. 1 in its courses for research design and data collection methods. The division of the research process in terms of a conceptual design, technical design, operationalisation, analysis plan, and sampling plan, has proved to be appealing for students of disciplines ranging from linguistics to bioengineering.

### Researching with MIR: noise reducing asphalt layers and quality of life

#### Research objective and research question

This example of the application of the MIR framework comes from a study about the effects of “noise reducing asphalt layers” on the quality of life (Vuye et al. 2016), a project commissioned by the City of Antwerp in 2015 and executed by a multidisciplinary research team of Antwerp University (Belgium). The principal researcher was an engineer from the Faculty of Applied Engineering (dept. Construction), supported by two researchers from the Faculty of Medicine and Health Sciences (dept. of Epidemiology and Social Statistics), one with a background in qualitative and one with a background in quantitative research methods. A number of meetings were held where the research team and the commissioners discussed the research objective (the ‘what’ and ‘why’).The research objective was in part dictated by the European Noise Directive 2002/49/EC, which forces all EU member states to draft noise action plans, and the challenge in this study was to produce evidence of a link between the acoustic and mechanical properties of different types of asphalt, and the quality of life of people living in the vicinity of the treated roads. While there was literature available about the effects of road surface on sound, and other studies had studied the link between noise and health, no study was found that produced evidence simultaneously about noise levels of roads and quality of life. The team therefore decided to test the hypothesis that traffic noise reduction has a beneficial effect on the quality of life of people into the central research. The general research question was, “to what extent does the placing of noise reducing asphalt layers increase the quality of life of the residents?”

#### Study design

In order to test the effect of types of asphalt, initially a pretest–posttest experiment was designed, which was expanded by several added experimental (change of road surface) and control (no change of road surface) groups. The research team gradually became aware that quality of life may not be instantly affected by lower noise levels, and that a time lag is involved. A second posttest aimed to follow up on this effect although it could only be implemented in a selection of experimental sites.

#### Instrument selection and design

Sound pressure levels were measured by an ISO-standardized procedure called the Statistical Pass-By (SPB) method. A detailed description of the method is in Vuye et al. (2016). No such objective procedure is available for measuring quality of life, which can only be assessed by self-reports of the residents. Some time was needed for the research team to accept that measuring a multidimensional concept like quality of life is more complicated than just having people rate their “quality of life” on a 10 point scale. For instance, questions had to be phrased in a way that gave not away the purpose of the research (Hawthorne effect), leading to the inclusion of questions about more nuisances than traffic noise alone. This led to the design of a self-administered questionnaire, with questions of Flanders Survey on Living Environment (Departement Leefmilieu, Natuur & Energie 2013) appended by new questions. Among other things, the questionnaire probed for experienced nuisance by sound, quality of sleep, effort to concentrate, effort to have a conversation inside or outside the home, physical complaints such as headaches, etc.

#### Sampling design

The selected sites needed to accommodate both types of measurements: that of noise from traffic and quality of life of residents. This was a complicating factor that required several rounds of deliberation. While countrywide only certain roads were available for changing the road surface, these roads had to be mutually comparable in terms of the composition of the population, type of residential area (e.g., reports from the top floor of a tall apartment building cannot be compared to those at ground level), average volume of traffic, vicinity of hospitals, railroads and airports, etc. At the level of roads therefore, targeted sampling was applied, whereas at the level of residents the aim was to realize a census of all households within a given perimeter from the treated road surfaces. Considerations about the reliability of applied instruments were guiding decisions with respect to sampling. While the measurements of the SPB method were sufficiently reliable to allow for relatively few measurements, the questionnaire suffered from considerable nonresponse which hampered statistical power. It was therefore decided to increase the power of the study by adding control groups in areas where the road surface was not replaced. This way, detecting an effect of the intervention did not solely depend on the turnout of the pre and the post-test.

#### Data analysis plan

The statistical analysis had to account for the fact that data were collected at two different levels: the level of the residents filling out the questionnaires, and the level of the roads which surface was changed. Because survey participation was confidential, results of the pre- and posttest could only be compared at aggregate (street) level. The analysis had to control for confounding variables (e.g., sample composition, variety in traffic volume, etc.), experimental factors (varieties in experimental conditions, and controls), and non-normal dependent variables. The statistical model appropriate for analysis of such data is a Generalised Linear Mixed Model.

#### Execution

Data were collected during the course of 2015, 2016 and 2017 and are awaiting final analysis in Spring 2017. Intermediate analyses resulted in several MSc theses, conference presentations, and working papers that reported on parts of the research.

## Discussion

In this paper we presented the Methodology in Interdisciplinary Research framework that we developed over the past decade building on our experience as lecturers, consultants and researchers. The MIR framework recognizes research methodology and methods as important content in the critical factor skills and competences. It approaches research and collaboration as a process that needs to be designed with the sole purpose to answer the general research question. For the conceptual design the team members have to discuss and agree on the objective of their communal efforts without squeezing it into one single discipline and, thus, ignoring complexity. The specific research questions, when formulated, contribute to (self) respect in collaboration as they represent and stand witness of the need for interdisciplinarity. In the technical design, different parts were distinguished to stimulate researchers to think and design research out of their respective disciplinary boxes and consider, for example, an experimental design with qualitative data collection, or a case study design based on quantitative information.

In our teaching and consultancy, we first developed a MIR framework for social sciences, economics, health and environmental sciences interdisciplinarity. It was challenged to include research in the design discipline of landscape architecture. What characterizes research in landscape architecture and other design principles, is that the design product as well as the design process may be the object of study. Lenzholder et al. (2017) therefore distinguish three kinds of research in landscape architecture. The first kind, “Research into design” studies the design product post hoc and the MIR framework suits the interdisciplinary study of such a product. In contrast, “Research for design” generates knowledge that feeds into the noun and the verb ‘design’, which means it precedes the design(ing). The third kind, Research through Design(ing) employs designing as a research method. At first, just like Deming and Swaffield (2011), we were a bit skeptical about “designing” as a research method. Lenzholder et al. (2017) pose that the meaning of research through design has evolved through a (neo)positivist, constructivist and transformative paradigm to include a pragmatic stance that resembles the pragmatic stance assumed in the MIR framework. We learned that, because landscape architecture is such an interdisciplinary field, the process approach and the distinction between a conceptual and technical research design was considered very helpful and embraced by researchers in landscape architecture (Tobi and van den Brink 2017).

Mixed methods research (MMR) has been considered to study topics as diverse as education (e.g., Powell et al. 2008), environmental management (e.g., Molina-Azorin and Lopez-Gamero 2016), health psychology (e.g., Bishop 2015) and information systems (e.g., Venkatesh et al. 2013). Nonetheless, the MIR framework is the first to put MMR in the context of integrating disciplines beyond social inquiry (Greene 2008). The splitting of the research into modules stimulates the identification and recognition of the contribution of both distinct and collaborating disciplines irrespective of whether they contribute qualitative and/or quantitative research in the interdisciplinary research design. As mentioned in Sect. 2.4 the integration of the different research modules in one interdisciplinary project design may follow one of the mixed methods designs. For example, we witnessed at several occasions the integration of social and health sciences in interdisciplinary teams opting for sequential modules in a sequential exploratory mixed methods fashion (e.g., Adamson 2005: 234). In sustainability science research, we have seen the design of concurrent modules for a concurrent nested mixed methods strategy (ibid) in research integrating the social and natural sciences and economics.

The limitations of the MIR framework are those of any kind of collaboration: it cannot work wonders in the absence of awareness of the necessity and it requires the willingness to work, learn, and research together. We developed MIR framework in and alongside our own teaching, consultancy and research, it has not been formally evaluated and compared in an experiment with teaching, consultancy and research with, for example, the regulative cycle for problem solving (van Strien 1986), or the wheel of science from Babbie (2013). In fact, although we wrote “developed” in the previous sentence, we are fully aware of the need to further develop and refine the framework as is.

## Outlook

The importance of the MIR framework lies in the complex, multifaceted nature of issues like sustainability, food security and one world health. For progress in the study of these pressing issues the understanding, construction and quality of interdisciplinary portfolio measurements (Tobi 2014) are pivotal and require further study as well as procedures facilitating the integration across different disciplines.

Another important strain of further research relates to the continuum of Responsible Conduct of Research (RCR), Questionable Research Practices (QRP), and deliberate misconduct (Steneck 2006). QRP includes failing to report all of a study’s conditions, stopping collecting data earlier than planned because one found the result one had been looking for, etc. (e.g., John et al. 2012; Simmons et al. 2011; Kampen and Tamás 2014). A meta-analysis on selfreports obtained through surveys revealed that about 2% of researchers had admitted to research misconduct at least once, whereas up to 33% admitted to QRPs (Fanelli 2009). While the frequency of QRPs may easily eclipse that of deliberate fraud (John et al. 2012) these practices have received less attention than deliberate misconduct. Claimed research findings may often be accurate measures of the prevailing biases and methodological rigor in a research field (Fanelli and Ioannidis 2013; Fanelli 2010). If research misconduct and QRP are to be understood then the disciplinary context must be grasped as a locus of both legitimate and illegitimate activity (Fox 1990). It would be valuable to investigate how working in interdisciplinary teams and, consequently, exposure to other standards of QRP and RCR influence research integrity as the appropriate research behavior from the perspective of different professional standards (Steneck 2006: p. 56). These differences in scientific cultures concern criteria for quality in design and execution of research, reporting (e.g., criteria for authorship of a paper, preferred publication outlets, citation practices, etc.), archiving and sharing of data, and so on.

Other strains of research include interdisciplinary collaboration and negotiation, where we expect contributions from the “science of team science” (Falk-Krzesinski et al. 2010); and compatibility of the MIR framework with new research paradigms such as “inclusive research” (a mode of research involving people with intellectual disabilities as more than just objects of research; e.g., Walmsley and Johnson 2003). Because of the complexity and novelty of inclusive health research a consensus statement was developed on how to conduct health research inclusively (Frankena et al., under review). The eight attributes of inclusive health research identified may also be taken as guiding attributes in the design of inclusive research according to the MIR framework. For starters, there is the possibility of inclusiveness in the conceptual framework, particularly in determining research objectives, and in discussing possible theoretical frameworks with team members with an intellectual disability which Frankena et al. labelled the “Designing the study” attribute. There are also opportunities for inclusiveness in the technical design, and in execution. For example, the inclusiveness attribute “generating data” overlaps with the operationalization and measurement instrument design/selection and the attribute “analyzing data” aligns with the data analysis plan in the technical design.

On a final note, we hope to have aroused the reader’s interest in, and to have demonstrated the need for, a methodology for interdisciplinary research design. We further hope that the MIR framework proposed and explained in this article helps those involved in designing an interdisciplinary research project to get a clearer view of the various processes that must be secured during the project’s design and execution. And we look forward to further collaboration with scientists from all cultures to contribute to improving the MIR framework and make interdisciplinary collaborations successful.
